# Do honey phytochemicals modulate forager aggression and the gut microbiome in the honey bee (*Apis mellifera* L.)?

**DOI:** 10.1242/bio.062233

**Published:** 2025-10-15

**Authors:** Wade A. Pike, Jaesylin Stephens, Mariah Donohue, Katsuri Rajandran, Erin D. Treanore, Abdallah Sher, Emily Croteau, Clare C. Rittschof

**Affiliations:** ^1^ Department of Entomology, University of Kentucky, Lexington, Kentucky 40546, USA; ^2^ Department of Biology, University of Kentucky, Lexington, Kentucky 40508, USA; ^3^ Department of Biological Sciences, Binghamton University, Binghamton, NY 13902, USA; ^4^ Department of Biology, Tufts University, Medford, MA 02155, USA

**Keywords:** Phytochemicals, Gut microbiome, Gut-brain axis, Pollinator, Aggression, Foraging strategies

## Abstract

Plant phytochemicals found in nectar impact bee learning and memory and plant pollination success. Especially for generalist pollinators, dietary changes that alter phytochemical consumption could be common sources of behavioral variation. For honey bee (*Apis mellifera* L.) foragers, a major potential change in phytochemical consumption occurs when individuals switch from collecting nectar from flowers to collecting honey from neighboring colonies, a phenomenon known as honey robbing. In this study we investigated whether phytochemicals dominant in honey compared to nectar act as a short-term trigger of robbing behaviors in honey bee, which include increased aggression. We fed forager honey bees sucrose diets containing different phytochemicals found in nectar and honey and tested aggression using a lab-based assay. We found no evidence that phytochemicals altered forager behavior. We also compared the microbiome composition for foragers fed different phytochemicals and again found no effects. Our results suggest that neither direct effects of neuroactive phytochemicals, nor indirect effects through the structure or function of the gut microbiome, trigger honey robbing behaviors.

## Teaser

**Summary:** Plant chemicals can impact the nervous system and behavior of pollinators. We found few effects of dietary plant chemicals on honey bee forager aggression and microbiome composition.

## INTRODUCTION

Plant phytochemicals, produced primarily for defense against insect predators (Dobler et al., 2011; Simmonds, 2001; Slavković and Bendahmane, 2023), are also found in floral nectar, where they are consumed by beneficial pollinators (Carlesso et al., 2021; Carter et al., 2006; Gao et al., 2010; Matsuura and Fett-Neto, 2015; Stevenson et al., 2017). Nectar phytochemicals impact cognitive and behavioral phenotypes like olfactory learning and resource fidelity (Couvillon et al., 2015; Wright et al., 2013), reward valuation (Hyman et al., 2006; Mustard et al., 2023), resource consumption (Nepi, 2014), and sociality (Gao et al., 2010; Narvaes and Martins de Almeida, 2014). Phytochemicals also promote antioxidant activity in various tissues (Ji-liang et al., 2020; Slavković and Bendahmane, 2023), provide muscle metabolic support (Nepi, 2014), alter gut microbiome diversity and gut pathogen tolerance (Bernklau et al., 2019; Geldert et al., 2021), and enhance immune system functions (Liao et al., 2017). Phytochemical effects in both nervous and non-nervous tissues can shape behavioral expression - for example, altering microbiome composition could influence the neuroactive metabolites it produces (Ahmed et al., 2022; O'Mahony et al., 2015; Yılmaz and Gökmen, 2020). Especially for generalist pollinators, dietary changes that alter phytochemical consumption could be common sources of behavioral variation.

For honey bee (*Apis mellifera* L.) foragers, a major potential change in phytochemical consumption occurs when individuals switch from collecting nectar from flowers to collecting honey from neighboring colonies, a phenomenon known as honey robbing (Free, 1954). The combination of diverse nectars and other biochemical processes in natural honey results in a chemical profile distinct from nectar (Alaerjani et al., 2022; Vazquez et al., 2021). Honey robbing is a risky strategy that occurs during periods of resource shortage, especially as winter approaches; ecological factors like floral resource availability, drought, and seasonality impact the likelihood that foragers will engage in honey robbing at particular times of the year (Downs and Ratnieks, 2000; Garbuzov et al., 2020; Kuszewska and Woyciechowski, 2014; Lindström et al., 2008; Rittschof and Nieh, 2021; Treanore et al., 2025). However, on an acute timescale, other more immediate cues are required to trigger the switch to robbing. For example, when foragers discover unprotected or weakly protected sources of honey (e.g. a weak or dead beehive, or honey exposed by beekeepers during honey harvesting; Peck and Seeley, 2019; Somerville et al., 2020), they often engage in robbing, and even redirect robbing behaviors towards other nearby colonies. This common link between honey exposure and robbing suggests there may be cues present in honey that trigger the behaviors. Here we investigate the hypothesis that forager feeding on phytochemicals common in honey trigger robbing and associated behaviors, either directly through actions on the nervous system, or indirectly, by altering the composition of the gut microbiome.

Robbing foragers exhibit distinct behavioral characteristics as they approach a victim hive. These include casting back and forth and seeking alternative entrances, presumably to avoid guards posted at the entrance of victim hives (Couvillon et al., 2008; Free, 1954). Previous work has demonstrated that robbing foragers also show temporarily elevated aggression compared to floral resource foragers (Grume et al., 2021), which could confer an advantage in conflicts with victim hive bees. Compared to behaviors like casting and alternative entrance seeking, which require field manipulations and observations, forager aggression can be measured at high throughput using a lab-based assay that quantifies the attack response towards a non-nestmate bee (Richard et al., 2012). Here we used this lab-based approach to assess if feeding on phytochemicals abundant in honey increases forager aggression compared to phytochemicals common in nectars or an untreated sucrose control. For a subset of these phytochemicals, we assessed impacts on the forager gut microbiome; because the microbiome produces critical neuromodulators (Zhang et al., 2022), changes in microbial community composition could be an indirect mechanism that induces robbing and associated behaviors like aggression (Nouvian et al., 2018).

We tested behavioral impacts of six plant phytochemicals based on their quantities in nectar compared to honey, and their known relationships to aggression in honey bees and other animals (Table 1). Two of these, quercetin and *p*-coumaric acid, are present in high concentrations in honey relative to nectar (Liao et al., 2017; Mao et al., 2013); quercetin is associated with queen-directed worker aggression (Gao et al., 2010). GABA, β-alanine, and taurine show relatively high abundances in nectar compared to honey. GABA is an inhibitory neurotransmitter that impacts motor function in honey bees (Mustard et al., 2020) and is associated with aggression in honey bees and other animals (Chandrasekaran et al., 2015; Hunt, 2007; Narvaes and Martins de Almeida, 2014). β-alanine is also associated with aggression in some invertebrate species (Bogo et al., 2019; Bouchebti et al., 2022; Jacobs, 1978). Taurine is a neuromodulator associated with motor function (Carlesso et al., 2021; Mustard et al., 2020), and it declines in concentration in the brains of honey bees exposed to aggression-inducing alarm pheromone (Chandrasekaran et al., 2015). Finally, we tested effects of L-proline because it is high in concentration in both nectar and honey (Carter et al., 2006; Iglesias et al., 2006; Saito et al., 2011), and it modulates activity patterns (Carter et al., 2006; Micheu et al., 2000). We predicted that *p-*coumaric acid and quercetin, and possibly L-proline, due to their high honey concentrations, would have positive impacts on forager aggression.

**Table 1.** Plant phytochemicals, their relative abundances in honey and nectar (based on previous studies), and whether the effects of these phytochemicals were tested for behavior and/or the microbiome in the current study

Phytochemical	Honey abundance	Nectar abundance	Behavior tested	Microbiome tested
β-alanine	Low (0.021 g/l)	High (0.024 g/l)	Yes	No
GABA	Low (0.013 g/l)	High (0.076 g/l)	Yes	Yes
Taurine	Low (0.009 g/l)	High (0.041 g/l)	Yes	No
*p*-coumaric acid	High (0.082 g/l)	Low (0.005 g/l)	Yes	Yes
L-proline	High (0.586 g/l)	High (0.461 g/l)	Yes	No
Quercetin	High (0.076 g/l)	Low (0.001 g/l)	Yes	No

β-alanine: (Carlesso et al., 2021; Hermosín et al., 2003; Iglesias et al., 2006); GABA: (Carlesso et al., 2021; Iglesias et al., 2006); taurine: (Carlesso et al., 2021; Pasantes-Morales et al., 1989); *p*-coumaric acid: (Bernklau et al., 2019; Liao et al., 2017); proline: (Bouchebti et al., 2022; Carter et al., 2006; Iglesias et al., 2006; Saito et al., 2011); quercetin: (Gao et al., 2010; Liao et al., 2017; Soto et al., 2013).

We chose two phytochemicals to evaluate impacts on the gut microbiome. We selected *p-*coumaric acid (high in honey, low in nectar) and GABA (high in nectar, low in honey) to compare to a sucrose control diet. *P*-coumaric acid has antimicrobial properties that could alter gut microbial composition (Liao et al., 2017; Mao et al., 2013), and GABA is strongly and canonically associated with aggression in many animal species (Abdou et al., 2006; De Almeida et al., 2005). Although this design does not enable us to determine how exactly the bacterial community could cause a change in forager aggression, an impact of phytochemical treatment on forager microbiome community composition would be the first step towards demonstrating a potential indirect effect of phytochemical feeding on the nervous system and behavior of robbing bees.

## RESULTS

### Phytochemicals dominant in honey have limited impacts on forager aggression

**Fig. 1.**
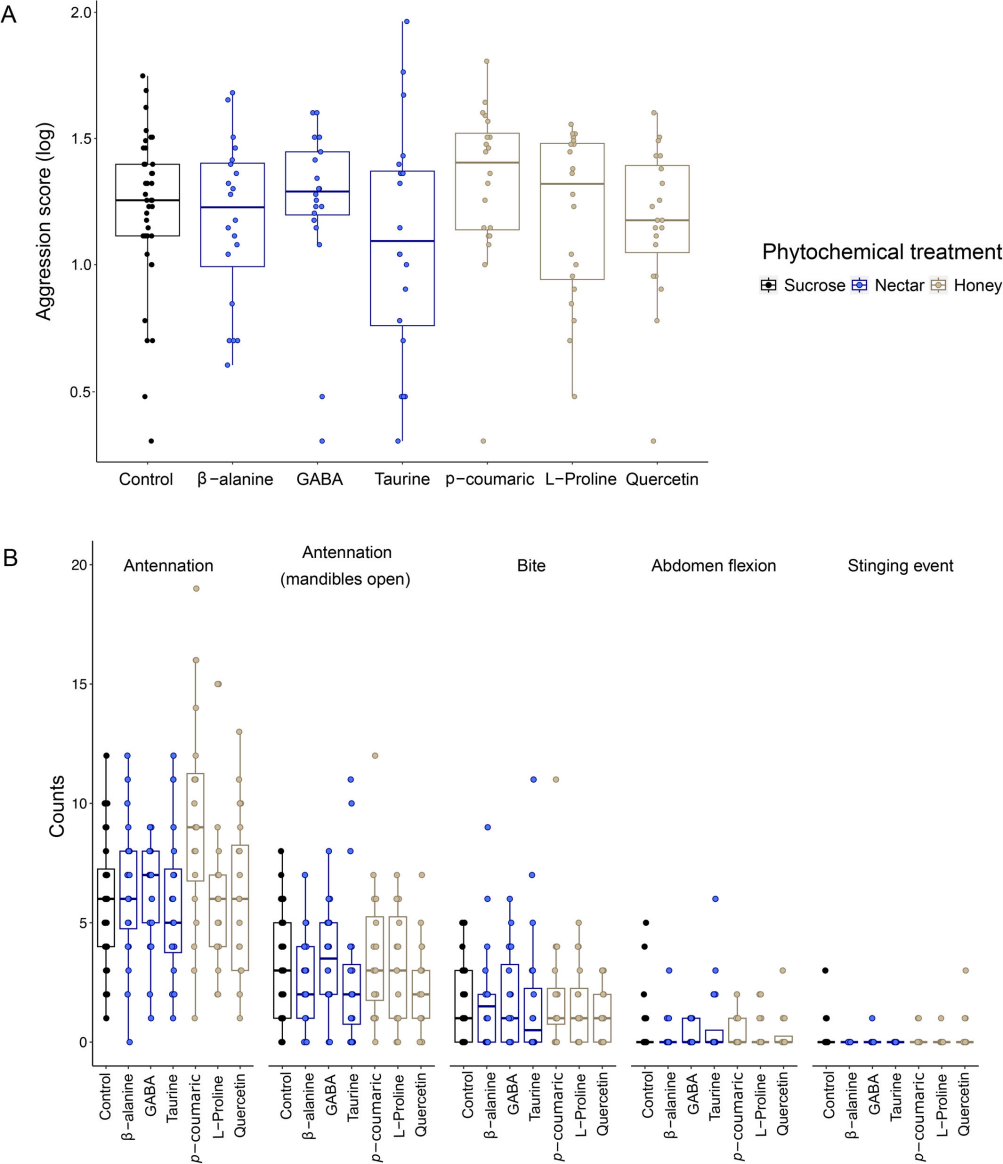
**Effects of phytochemical treatment on forager aggression.** (A) The log-transformed aggression score weights tallies of aggressive behaviors by severity and (B) the effects of phytochemical treatment on counts of aggressive behaviors. We fed bees one of seven diet treatments: a control (black dots and boxes, 40% sucrose), one of three phytochemicals with high concentrations in nectar (blue dots and boxes, presented in 40% sucrose), or one of three phytochemicals with high concentrations in honey (gold dots and boxes, presented in 40% sucrose, see also Table 1). Each dot represents a group of three bees challenged by a non-nestmate intruder (*N*=40 for control, *N*=20 for each phytochemical). Boxplots show medians and interquartile ranges, and whisker tips are 1.5*IQR.

In a linear mixed model with aggression score as a response variable, phytochemical treatment and fanning behavior (see Materials and Methods) as fixed effects, and colony identity as a random effect, we found no effect of phytochemical treatment on forager aggression (phytochemical treatment: X^2^_6_*=*11.4, *P*=0.08, fanning behavior: X^2^_1_*=*31.9, *P*<0.0001, Fig. 1A). Post-hoc pairwise comparisons between each phytochemical and the control also showed no significant effects (Table S2). In a similar analysis that grouped phytochemicals by their relative concentration in nectar versus honey (high in nectar: ß-alanine, GABA, and taurine, and high in honey: *p*-coumaric acid, L-proline, and quercetin), aggression score was not significantly different for phytochemicals that are dominant in nectar versus honey (linear mixed model, honey or nectar: X^2^_1_=2.77, *P*=0.10, Fig. 1A).

We also tested the effects of phytochemical treatment on each aggressive behavior (see Materials and Methods). For two low-level aggressive behaviors, antennation with and without mandibles open, we found a significant effect of phytochemical treatment (Fig. 1B, Table S3, antennation: phytochemical treatment: X^2^_6_*=*29.1, *P*<0.001; antennation with mandibles open: phytochemical treatment: X^2^_6_*=*16.9, *P*=0.01). Post-hoc pairwise comparisons for antennation showed that *p*-coumaric acid-treated bees showed significantly more antennation compared to all other phytochemical treatments, including the control sucrose. For antennation with mandibles open, *p*-coumaric acid-treated bees showed significantly more behaviors than quercetin-treated bees; no other comparisons were significant, including *p*-coumaric acid versus control. Biting, abdomen flexion, and stinging showed no significant differences as a function of treatment (biting: X^2^_6_=2.0, *P*=0.9, abdomen flexion: X^2^_6_=1.1, *P*=1.0, stinging: X^2^_6_=7.6, *P*=0.3).

### Phytochemicals do not affect gut microbiome composition or diversity

**Fig. 2.**
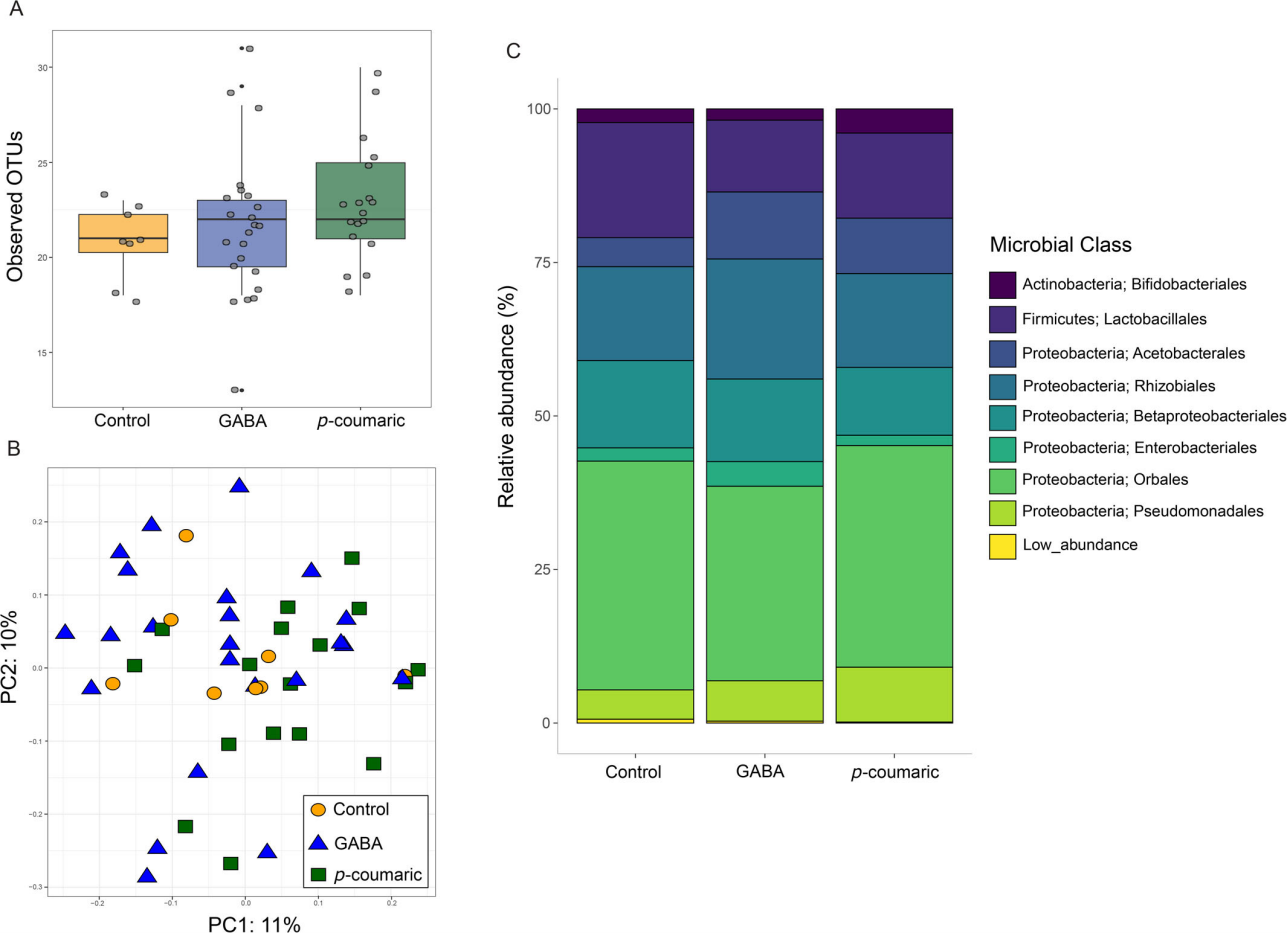
**Impacts of phytochemical treatment on gut microbiome composition.** (A) Number of observed OTUs, a measure of alpha diversity. Each dot represents a sample (*N*=10 control, *N*=26 GABA, *N*=22 *p*-coumaric acid). Boxplots show the median and interquartile range, with whiskers reaching 1.5*IQR. Black dots indicate outlier samples. (B) Principal component analysis of beta diversity (UniFrac) with each symbol representing a sample. (C) Mean relative abundance of various microbial classes as a function of phytochemical treatment.

After filtering, we generated a mean of 9085 per sample (3810 to 17,159). The most abundant microbial phylum was Proteobacteria (82% of reads per sample), followed by Firmicutes (15%) and Actinobacteria (3%). The most abundant microbial classes included Orbales (Proteobacteria phylum; 35%), Rhizobiales (Proteobacteria phylum; 17%), Lactobacillales (Firmicutes phylum; 15%), and Betaproteobacteriales (Proteobacteria phylum; 13%). Phytochemical treatment did not have a significant effect on microbiome diversity (Fig. 2, Tables S4, S5), and analysis of composition of microbiomes (ANCOM) did not detect any significant differences in microbial composition across phytochemical treatment groups.

## DISCUSSION

We evaluated the hypothesis that honey-dominant phytochemical exposure triggers aggressive honey robbing in foraging honey bees. We found that exposure to phytochemicals dominant in honey versus nectar (or an untreated control) had limited impacts on aggression, suggesting few direct effects of ingesting phytochemicals on aggression-related nervous system function in forager bees. Furthermore, more persistent exposure to two phytochemicals, GABA (dominant in nectar) and *p*-coumaric acid (dominant in honey), did not affect gut microbiome content and diversity relative to a sucrose control diet, suggesting few indirect effects of phytochemicals on behavior through microbiome activity.

The timing and quantity of phytochemical consumption could greatly impact behavioral outcomes, and we did not investigate this possibility in the current study. Both short- and long-term phytochemical exposure contexts are relevant for pollinators (Palmer-Young et al., 2019; Si et al., 2005; Wright et al., 2013). Here we allowed bees to consume phytochemicals *ad libitum* for about 16 h before measuring aggression. We chose this timeframe based on previous studies that used this same aggression assay to demonstrate behavioral effects of other dietary constituents and neuroactive compounds (Li-Byarlay et al., 2014; Rittschof et al., 2018). This is a longer timeframe than previous studies showing learning and memory effects of phytochemicals (Marchi et al., 2021; Wright et al., 2013), and it exceeds the minimum timeframe required for a colony to shift to honey robbing (Grume et al., 2021; Napier et al., 2023). However, this could be a realistic exposure timeframe for a nectar forager that persists visiting one plant species over multiple trips or even across multiple days due floral constancy (Gruter and Ratnieks, 2011), or a robbing forager that demonstrates high persistence at a victim hive (Lindström et al., 2008; Peck and Seeley, 2019). It is possible that over the timeframe of our experiment, bees acclimated to the dietary phytochemicals, eliminating any observable behavioral effects. Notably, we chose a longer exposure time for the microbiome study (5 days) based on a previous study showing phytochemical effects (including for *p*-coumaric acid) on microbial diversity and relative abundance (Geldert et al., 2021). That previous study did find evidence that longer exposure (6-day versus 3-day exposure) led to some reversion to baseline community composition, suggesting it is possible that the microbial community acclimated to phytochemical treatment over our 5-day timeframe. Future studies could assess how bees habituate or become sensitized to phytochemical exposure over time; such effects are possible and could impact the adaptive explanations for phytochemical presence in nectar.

In our experiments, we applied phytochemicals one at a time. However, some nectars, and certainly honey which is a combination of many nectars, contain combinations of phytochemicals. Dietary phytochemicals can have synergistic or antagonistic effects on the consumer (Chen et al., 2022; Hemingway et al., 2024). For example, Marchi et al. (2021) demonstrated synergistic effects of caffeine and arginine on memory retention in honey bees; combinations of phytochemicals also act synergistically in the gut (Palmer-Young et al., 2017). In terms of the phytochemicals used in our study, taurine and β-alanine act in combination to affect mouse behavior (Murakami and Furuse, 2010). In rats, *p*-coumaric acid activates GABA receptors, demonstrating possible synergy between these two compounds (Scheepens et al., 2014). For simplicity, we also tested a single concentration of each phytochemical. However, concentrations in nectar and honey likely vary due to factors like nectar sugar concentration or the age of stored honey (Al–Sherif et al., 2017; Iglesias et al., 2006; Nicolson, 2022). The extent of variation in nectar and honey phytochemical composition is largely unknown; future work could address this variation, as well as its neurobiological and behavioral implications.

As expected, based on many previous studies in *A. mellifera* (Raymann and Moran, 2018), we observed a stable set of core microbiota in our samples. There are eight core phylotypes that make up approximately 99% of the bacteria in the gut microbiome of all adult worker bees (Kwong and Moran, 2016; Moran et al., 2012). Our control group had the largest percentage of low abundance bacteria, suggesting phytochemical feeding may decrease bacterial diversity, but this pattern was not statistically significant. Core microbiota should encompass microbes necessary for nutrient acquisition, which would explain their resiliency against external variables like host diet. Since a forager-aged honey bee's natural diet mostly consists of carbohydrates from nectar and honey (Paoli et al., 2014), carbohydrate-metabolizing bacteria dominate honey bee guts, which is consistent with our results (Han et al., 2024; Kwong et al., 2017). Geldert et al. (2021) found impacts of phytochemical feeding (including *p*-coumaric acid) on honey bee gut bacterial richness, gut fungal Shannon diversity, and beta diversity for both microbe types; other recent studies have also found significant effects of phytochemicals on the microbiome (Saelao et al., 2020; Taylor et al., 2019). One substantial difference between our work and these previous studies is worker specialization and age; previous studies sampled 7–10-day-old workers, which are younger than our focal bees, typically hive-bound, and may still consume pollen if they are involved in nursing activities (Naiem et al., 1999). In our caged study, foragers also lacked exposure to the physical hive environment and other adult workers like nurse bees, which share nutritional secretions; these factors are both known to influence microbial community composition (Powell et al., 2014). In addition, we conducted our study using the entire honey bee gut, while many studies choose to focus on only the hindgut, rectum, or ilium because these various sections contain unique and differing proportions of bacteria (Raymann and Moran, 2018; Smutin et al., 2022). Finally, while we successfully categorized all the bacteria in our dataset into their respective phyla, we could not identify strain-level variation in the gut microbiome, which could limit our ability to detect phytochemical treatment effects. Overall, the extent to which the microbiome changes in response to environmental factors remains unresolved in honey bees (Jia et al., 2016; Ludvigsen et al., 2015; Wang et al., 2020; Yun et al., 2018), but it is possible that the microbiome of foraging bees, who consume almost exclusively carbohydrates, is resilient to dietary variation.

## MATERIALS AND METHODS

### Honey bee sources and collections

We conducted this experiment in August and September 2022, which is within the seasonal timeframe in our area when robbing and associated behaviors are common (Treanore et al., 2025). Focal honey bee workers originated from colonies on a University of Kentucky research farm in Lexington, KY, USA (38.127, −84.511). We used two mature, full-sized colonies headed by naturally mated queens. Colonies were derived from commercial stocks (advertised as Italian, Carniolan, and Russian hybrid) interbred with the local population, and were maintained according to standard beekeeping practices as outlined by the Honey Bee Health Coalition. Colonies were healthy (low or undetected *Varroa* mite levels) and received no diet supplements or *Varroa* mite treatment during the experiment.

While colonies were actively foraging, we collected workers from the entrance using a portable insect vacuum (formerly Bioquip, Rancho Dominguez, CA, USA). These focal bees were mostly foragers, but there may have also been some guards, fanners, or undertakers (Butler and Free, 1952; Seeley, 1982) distributed randomly across our treatment groups. We placed bees in plexiglass cages (8 cm×9.5 cm×6.5 cm) with 30 bees each. Two cages from each colony were haphazardly assigned to each diet treatment (*N*=4 cages per treatment total). Within 10–15 min of collection, we provided caged bees *ad libitum* diet treatments in feeders, which were 1.5 ml microcentrifuge tubes (Eppendorf, Enfield, CT, USA) with two small feeding holes drilled into the bottom (Preston et al., 2019). We kept cages in an incubator (34°C) overnight and assayed aggression the following day (see below). Due to difficulties obtaining quercetin, we tested five phytochemicals and a control sucrose diet simultaneously in August 2022 and then tested quercetin in September 2022 with an additional control diet treatment in September in case there were season-related shifts in robbing behaviors and/or aggression (Garbuzov et al., 2020).

### Phytochemical treatments

To prepare diets, we added one phytochemical to a 40% (m/m) sucrose solution. 40% sucrose without any phytochemical added served as a control. We added phytochemicals in naturally occurring concentrations found in nectar (see also Table 1; GABA: 0.73 mM, β-alanine: 0.27 mM, taurine: 0.32 mM, L-proline: 4.0 mM) or honey (*p*-coumaric acid: 0.5 mM, quercetin: 0.25 mM). Product numbers and sources can be found in Table S1. Because *p*-coumaric acid and quercetin are insoluble in water, we first dissolved them in dimethyl sulfoxide (DMSO, Sigma-Aldrich, D8418-500ML). Approximately 550 µl of *p*-coumaric acid solution and 250 µl of quercetin solution were added to 1 liters of sucrose solution to achieve the above concentrations, resulting in minimal DMSO exposure (Liao et al., 2017).

### Aggression assays

We tested aggression after approximately 16 h of *ad libitum* diet exposure. Although this is a longer timeframe than, e.g. a single bout of drinking honey or nectar, the time frame matches previous studies showing dietary effects of neuroactive compounds on aggression (Rittschof et al., 2018). Moreover, foragers typically visit only a single plant species in a single trip (Gruter et al., 2011) and can persist foraging on a single flower species across multiple days (Free, 1963); a colony that switches to honey robbing often continues for several hours or until foraging ends for the day. Therefore, the 16 h treatment timeframe reasonably captures potential phytochemical exposure under floral and robbing foraging conditions.

We evaluated aggression using an ‘intruder assay’ (originally designed by Breed, 1983), which measures the aggressive response of a small group of focal bees (in this case, our diet-treated bees) to a non-nestmate ‘intruder’ bee (Harrison et al., 2019; Li-Byarlay et al., 2014; Rittschof, 2017). This assay was originally designed to measure the defensive behaviors of guard bees, but previous studies show that it detects variation in individual aggression across a wide variety of experimental contexts and worker specialists (Grume et al., 2021; Harrison et al., 2019; Li-Byarlay et al., 2014; Richard et al., 2012; Rittschof, 2017; Rittschof et al., 2015). Here we are using it to infer variation in forager aggression. To set up the small groups, we transferred three diet-treated bees from the same plexiglass cage into a sealed Petri dish (100 mm×20 mm) and allowed them 2 h to acclimate to their new environment. During this time, they were provided a feeder tube containing their same diet treatment. For the August assays, we set up *N*=20 such groups each for GABA, β-alanine, taurine, *p*-coumaric acid, L-proline, and control diets (*N*=120 total assays). For September, we set up 40 assays, *N*=20 for quercetin and *N*=20 for the control diet. This resulted in *N*=160 total assays over the course of the experiment. At the time we set up the small groups, we visually estimated the volume of diet remaining in the feeding tubes from the overnight cages (*N*=4 per diet treatment, *N*=8 for the sucrose control); we found no significant differences in diet consumption (*F*_6,23_=2.2, *P*=0.08, Fig. S1).

To initiate the assay and observations, blinded observers applied a small dot of acrylic paint (Testors, Rockford, IL, USA) to the thorax of an ‘intruder bee’, which was a worker collected from a colony different from the focal bee source. The intruder was placed in the Petri dish with the focal group, and we recorded aggressive behaviors exhibited by focal bees towards the intruder bee for 3 min. Aggressive behaviors include antennation, where a focal bee uses her antennae to smell an intruder bee, antennation with mandibles open, biting events on the legs, body, and antennae, abdomen flexion, where a focal bee climbs on top of the intruder and curls her abdomen under as if to sting, and stinging, where a focal bee extrudes her stinger and attempts to penetrate the cuticle of the intruder (Grume et al., 2021; Li-Byarlay et al., 2014; Richard et al., 2012; Rittschof et al., 2018). Following the 3 min assay, focal bees were released.

We used the tallies of aggressive behaviors to calculate an aggression score. We multiplied tallies of each behavior by a fixed value that indicates the severity of the behavior (1: antennation, 2: antennation with open mandibles, 3: biting, 4: abdomen flexion, 5: stinging attempts) and then summed these totals. This scale has been used extensively in previous studies (Grume et al., 2021; Richard et al., 2008). It also reflects the typical progression of defensive interactions observed between focal bees and an intruder at the colony entrance (Grume et al., 2021).

### Microbiome collections and sample processing

We conducted microbiome studies on a second, separate set of bees collected from two colonies during July 2022. One of the colonies was included in both the behavior and microbiome studies, while the second colony was unique to the microbiome study. We chose two phytochemicals to evaluate impacts on the gut microbiome. We selected *p-*coumaric acid (concentrated in honey, low in nectar) and GABA (concentrated in nectar, low in honey) to compare to a sucrose control diet. Bees were collected and housed as described above for the aggression assays, but they received a longer diet exposure of 5 days to allow for any changes in microbiome growth associated with phytochemical exposure.

Following the 5 days of *ad libitum* diet treatment, we flash-froze the bees and stored them at −80°C. For gut extraction, honey bees were thawed on ice, then washed with 95% ethanol. Using sterile tools, the head, wings and legs were cut off leaving the thorax and abdomen intact. Sterile forceps were used to pinch the top of the thorax and bottom of the abdomen. The bottom of the abdomen was pulled while holding the thorax steady, eviscerating the intact gut from the rest of the bee. Guts were placed in 1.5 ml tubes and frozen at −20°C until DNA extraction.

We generated microbial DNA sequence data from 58 individual gut samples (*N*=10 sucrose control, *N*=26 GABA, *N*=22 *p*-coumaric acid). DNA was extracted using the Quick-DNA Fecal/Soil Microbe MiniPrep Kit (cat. no. D6010, Zymo Research, Irvine, CA, USA) following the manufacturer protocol. The V4 region of the 16S rRNA gene was amplified using the polymerase chain reaction (PCR) from each sample with dual-indexed primers (Kozich et al., 2013). PCRs were conducted in 20 μl volumes and contained 10 μl of 2X GoTaq PCR Master Mix (Promega), 1 μl of each of the 10 μM forward and reverse primers, 2 μl of 1-25 ng DNA template, and 6 μl of PCR-grade dH**_2_**O. PCR amplification was performed in a thermal cycler with a 3-min initial denaturation at 95°C followed by 34 cycles of 45-s at 95°C, 1-min at 50°C, 3-min at 72°C, and a final extension of 2-min at 72°C. Size and quality of PCR products were confirmed using 1 1% agarose gel. DNA was quantified using a Qubit fluorometer (Thermo Fisher Scientific). Libraries were then normalized to ∼1 ng/μl by diluting with PCR-grade dH_2_O. Finally, samples were pooled and submitted to the UK HealthCare Genomics Core for sequencing on an Illumina MiSeq flowcell using a v2 reagent kit and 250 bp paired end reads. Sequences were demultiplexed using the Illumina MiSeq pipeline.

### Statistical analysis

We analyzed behavioral data using R v 2021.09.0. Significance of linear mixed model main effects were assessed using the anova function, and post-hoc pairwise comparisons of planned contrasts (each diet treatment compared to the sucrose control) were conducted using emmeans. We constructed figures using the R package ggplot2.

Because aggression score was over-dispersed and skewed, we log-transformed these values to fit a linear mixed model. Prior to analyzing phytochemical treatment effects, we first assessed differences in aggression for our control groups measured in August and September. Finding no differences in aggression score (two-tailed *t*-test, t_32.4_=1.72, P=0.09), we pooled control groups and conducted all further analyses on all phytochemical treatments together. During the aggression assays we tallied wing fanning presence or absence. When bees fan their wings, they stand still, expose their Nasanov gland, and emit an aggregation pheromone (Bortolotti and Costa, 2014); previous studies have shown a negative correlation between wing fanning and quantity of aggressive behaviors displayed (Carr et al., 2020). We performed two-tailed *t*-tests for effects of observer (t_151.5_=−1.3, *P*=0.18) and wing fanning (t_157.5_=5.6, *P*<0.0001) and decided to include fanning (but not observer) as a fixed effect in our final models of aggression score. Because samples from a single colony are socially and genetically non-independent, we included colony identity as a random effect in all analyses. Thus, our final linear mixed model for aggression score included phytochemical treatment and fanning as fixed effects and colony identity as a random effect. We verified the quality of model fit by examining the distribution of model residuals.

In addition to testing the effects of individual phytochemical treatments on aggressive behavior, we grouped phytochemicals according to their relative concentrations in honey versus nectar. *P*-coumaric acid and quercetin are high in honey relative to nectar, while β-alanine, GABA, and taurine are all relatively high in nectar (Table 1); for this analysis, we considered L-proline high in honey, even though it is present in substantial quantities in nectar as well. As above, we constructed a linear mixed model with aggression score as a response variable but used honey versus nectar groupings as the fixed effects instead of phytochemical identity individually.

We also analyzed the effects of phytochemical treatment on each individual aggressive behavior (five total behaviors). For antennation and antennation with mandibles open, we performed generalized linear mixed models (Poisson, log-link family) with phytochemical treatment and fanning as fixed effects, and colony identity as a random effect. As above, we performed post-hoc pairwise analyses of planned contrasts (each phytochemical relative to the sucrose control) for these models. For all other behaviors, which occur at a relatively low frequency, we compared phytochemical treatments using non-parametric Kruskal–Wallis tests.

For the microbiome analyses, Illumina reads were filtered and processed using the DADA2 pipeline (Callahan et al., 2016) in QIIME2 v.2018.11 (Bolyen et al., 2019). Amplicon sequence variants (ASVs) were classified using the SILVA reference database version 132 (Quast et al., 2013). Samples with less than 1000 reads (*N*=7) were excluded from downstream analyses, leaving 51 samples total pooled across two colonies (*N*=9 control, *N*=23 GABA, *N*=19 *p*-coumaric acid). To determine the minimum number of reads required for accurate estimations of gut microbiome diversity, we generated a rarefaction curve (Fig. S2); the curve plateaued at about 500 reads per sample, so we performed diversity analyses with at the sequencing depth of the lowest sample (3953 reads).

We measured alpha diversity using Faith's phylogenetic diversity (PD), a phylogenetic generalization of species richness (Chao et al., 2010), and observed OTUs, a simple count of the number of unique ASVs (Quast et al., 2013). The effects of phytochemical treatment on Faith's PD and observed OTUs were assessed using pairwise Kruskal–Wallis tests with Benjamini and Hochberg correction (Benjamini and Hochberg, 1995). We measured beta diversity using four standard metrics – Bray-Curtis, Jaccard, unweighted UniFrac, and weighted UniFrac – all of which leverage distinct algorithms for calculating differences in microbiome composition between samples (Estaki et al., 2020; Lozupone and Knight, 2005). For example, Bray-Curtis and weighted UniFrac consider ASV abundance, while Jaccard and unweighted UniFrac only consider presence/absence; both UniFrac metrics take phylogenetic relatedness and branch lengths into account. Previous work has shown that UniFrac metrics are more sensitive to divergence in ancient microbial clades (i.e. long branch lengths), while taxonomic metrics (Bray-Curtis and Jaccard) are better at detecting nascent community divisions (Donohue et al., 2022; Sanders et al., 2014). The effects of phytochemical treatment on each measure of beta diversity were tested using pairwise PERMANOVA tests with Benjamini-Hochberg correction.

Finally, we determined whether variation in relative ASV abundance was associated with phytochemical treatment. To do this, we used ANCOM. ANCOM has two outputs – F-statistics, which estimate effect size, and W-statistics, which quantify shifts in the number of reads attributed to a given ASV relative to others in the dataset; generally, the higher the F- and W-statistic, the more significant an ASV's differential abundance (Mandal et al., 2015).

### Conclusion

Dietary phytochemicals are critical for bee physiology and behavior and thus shifts in resource consumption could be a common source of behavioral variation among individuals. Neuroactive phytochemicals prevalent in honey do not appear to cause increased aggression in forager bees compared to nectar-dominant phytochemicals or a sucrose control. Honey-dominant phytochemicals also have limited indirect effects on nervous system function through changes to the gut microbiome community. We thus have little evidence that phytochemicals prevalent in honey explain why honey exposure triggers robbing behaviors in forager bees. Although it is possible that the timing of phytochemical exposure, phytochemical dosage, or synergistic effects of phytochemicals in combination could be required to trigger robbing behaviors, other cues and experiences may also play a role. For example, a recent study suggests that physical conflict with conspecifics at contested food resources may induce increased forager aggression and other robbing behaviors (Napier et al., 2023). Future work should also examine how immediate robbing triggers like exposure to honey and fights with conspecifics act in combination with seasonal and ecological conditions like floral resource shortages and drought to modulate the expression of robbing behaviors in honey bees.

## Supplementary Material

10.1242/biolopen.062233_sup1 Supplementary information
