# Supplementary material for: Do honey phytochemicals modulate forager aggression and the gut microbiome in the honey bee (Apis mellifera L.)?
Source: Biol Open. 2025 Oct 15;14(10):bio062233. doi: 10.1242/bio.062233 (PMC12570152; doi:10.1242/bio.062233)
Supplement: Supplementary information [file biolopen-14-062233-s1.pdf]

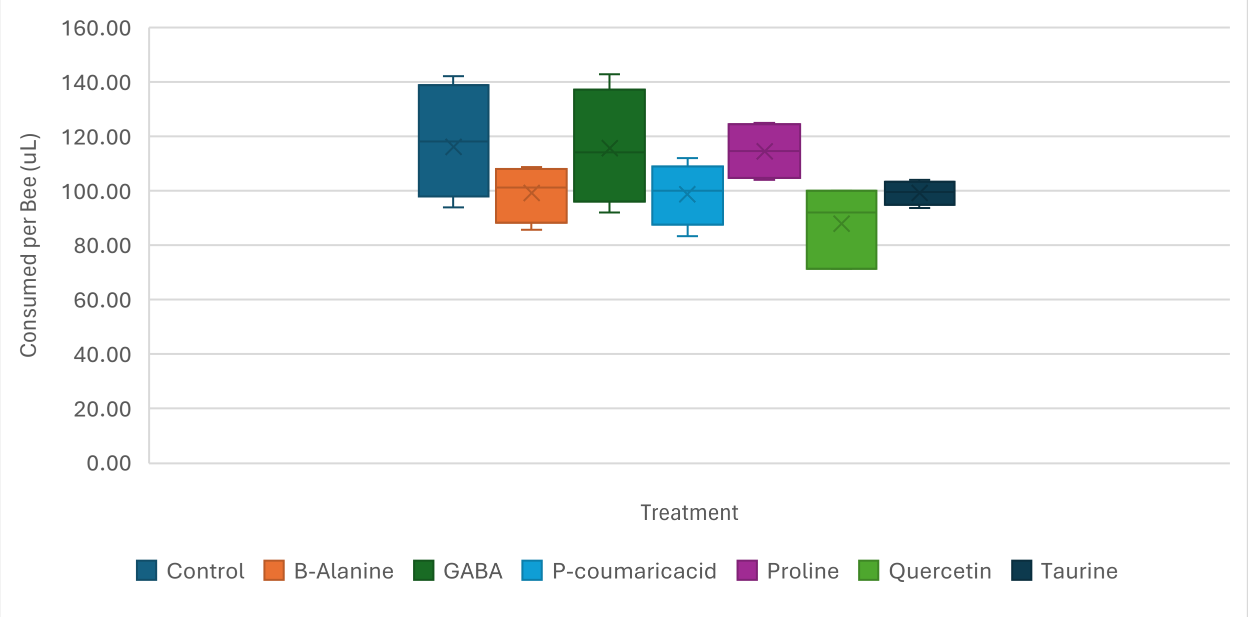

Fig. S1. Estimated diet mixture consumed per bee overnight prior to behavioral testing. Data show medians, interquartile ranges, and 1.5*IQR for N=4 boxes of bees per diet treatment.

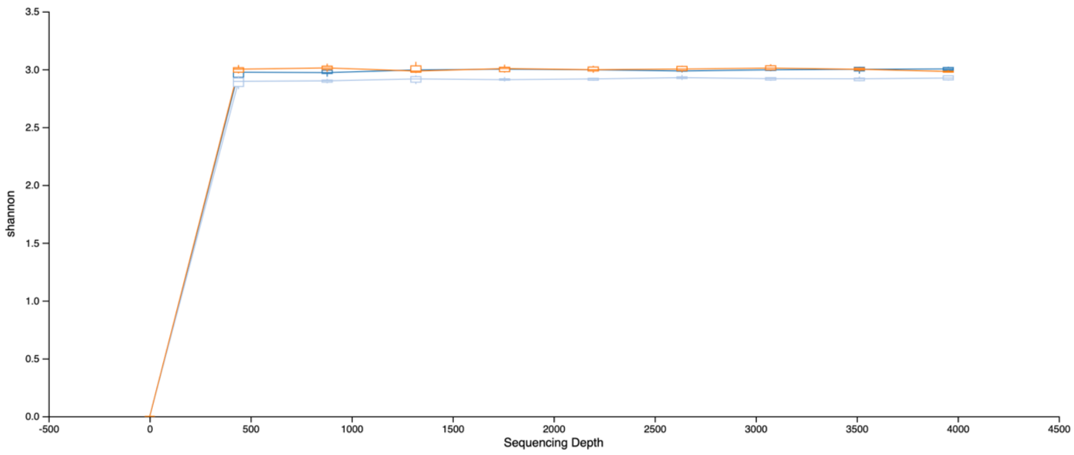

Fig. S2. Rarefaction curve shows the minimum number of reads used to generate Shannon Index diversity metrics. Samples with more than 1,000 reads (n=51) were used for analyses.

Biology Open • Supplementary information

Table S1. Phytochemical product numbers and

Chemical	Manufacturer	Item Number
GABA	Sigma-Aldritch	A2129-25G
B-alanine	Sigma-Aldritch	5160-50G
Taurine	Sigma-Aldritch	T0625-25G
L-proline	Sigma-Aldritch	P5607-25G
p-coumaric acid	Sigma-Aldritch	C9008-10G
Quercetin	CHEM-IMPEX	3896

Table S2. Pairwise comparisons of aggression score mean for phytochemical treatments relative to sucrose control.

Phytochemical	Estimate	T	P
ß-alanine	0.14	1.39	0.81
GABA	0.04	0.39	1.00
Taurine	0.21	2.10	0.36
p-coumaric acid	-0.10	-1.01	0.95
Proline	0.02	0.17	1.00
Quercetin	0.13	1.33	0.84

Table S3. Post-hoc pairwise phytochemical treatment comparisons of mean counts of antennation and antennation with mandibles open behaviors. Significant contrasts are in bold.

	Antennation		Antennation with mandibles open	
Contrast	Estimate	P	Estimate	P
Control - (B-alanine)	-0.015685	0.99999933	0.35156558	0.37251992
GABA - (B-alanine)	-0.0202311	0.99999861	0.36858427	0.4263813
GABA - Control	-0.0045461	1	0.01701869	0.99999983
(P-coumaric) - (B-alanine)	**0.4272531**	**0.00422347**	0.48458137	0.12502792
(P-coumaric) - Control	**0.44293808**	**0.00010317**	0.13301579	0.97384817
(P-coumaric) - GABA	**0.44748415**	**0.00227268**	0.1159971	0.99351922
Proline - (B-alanine)	0.01183357	0.99999995	0.38642754	0.40789583
Proline - Control	0.02751855	0.99998039	0.03486196	0.9999893
Proline - GABA	0.03206462	0.99997965	0.01784327	0.99999991
Proline - (P-coumaric)	**-0.4154195**	**0.00603475**	-0.0981538	0.99770747
Quercetin - (B-alanine)	-0.0261342	0.99999356	-0.1301761	0.99600752
Quercetin - Control	-0.0104492	0.99999994	-0.4817416	0.098246
Quercetin - GABA	-0.0059032	1	-0.4987603	0.13601927
Quercetin - (P-coumaric)	**-0.4533873**	**0.00193189**	**-0.6147574**	**0.02577729**
Quercetin - Proline	-0.0379678	0.99994569	-0.5166036	0.12878883

Taurine - (B-alanine)	-0.096217	0.98972781	0.20081888	0.94476688
Taurine - Control	-0.080532	0.99231716	-0.1507467	0.96626264
Taurine - GABA	-0.0759859	0.9972622	-0.1677654	0.96580582
Taurine - (P-coumaric)	**-0.5234701**	**0.00021876**	-0.2837625	0.68425643
Taurine - Proline	-0.1080505	0.98204623	-0.1856087	0.95178945
Taurine - Quercetin	-0.0700827	0.99826255	0.33099493	0.65311942

Table S4. Kruskal-Wallis tests show no significant effect of phytochemical treatment on gut microbiome alpha diversity.

Alpha Diversity Metric	Comparison	H-value	p-value	q-value (adjusted p-value)
Faith's PD	Control vs. GABA	1.834	0.18	0.26
	Control vs. PCOU	0.014	0.91	0.91
	GABA vs. PCOU	2.063	0.15	0.26
Observed OTUs	Control vs. GABA	0.276	0.60	0.60
	Control vs. PCOU	2.538	0.11	0.33
	GABA vs. PCOU	1.374	0.24	0.36

Table S5. PERMANOVA tests show no significant effect of phytochemical treatme nt on gut microbiome beta diversity after controlling for multiple comparisons.

Beta Diversity Metric	Comparison	pseudo-F	p-value	q-value (adjusted p-value)
Bray-Curtis	Control vs. GABA	1.282	0.22	0.61
	Control vs. PCOU	0.631	0.79	0.79
	GABA vs. PCOU	1.030	0.41	0.61
Jaccard	Control vs. GABA	1.133	0.30	0.30
	Control vs. PCOU	1.797	**0.02***	0.05
	GABA vs. PCOU	1.278	0.19	0.29
Unweighted UniFrac	Control vs. GABA	2.566	**0.04***	0.11
	Control vs. PCOU	1.600	0.15	0.22
	GABA vs. PCOU	0.937	0.44	0.44
Weighted UniFrac	Control vs. GABA	1.651	0.15	0.44
	Control vs. PCOU	0.707	0.61	0.61
	GABA vs. PCOU	0.831	0.52	0.61

*** <0.05**
